# Fava bean plant performance and fertility dynamics in Mars regolith simulant-based substrates for space farming

**DOI:** 10.3389/fpls.2025.1676285

**Published:** 2025-10-13

**Authors:** Antonio Giandonato Caporale, Roberta Paradiso, Nafiou Arouna, Chiara Amitrano, Silvia Tagliamonte, Manuela Flavia Chiacchio, Paola Vitaglione, Stefania De Pascale, Paola Adamo

**Affiliations:** Department of Agricultural Sciences, University of Naples Federico II, Piazza Carlo di Borbone, Naples, Italy

**Keywords:** *Vicia faba* L., Bioregenerative Life Support Systems (BLSSs), *in situ* resource utilization (ISRU), regolith simulant MMS-1, legumes

## Abstract

**Introduction:**

Plants are promising bioregenerators for long-term space missions. However, space cultivation will require fertile substrates based on *in-situ* available materials.

**Methods:**

We assessed the response of fava bean (*Vicia faba* L. cv. 'Sfardella') to glasshouse cultivation on six substrates: pure MMS-1 Mars regolith simulant (R100), MMS-1 amended with green compost 70:30 v:v (R70C30), pure fluvial sand (S100), sand mixed with compost 70:30 v:v (S70C30), sandy-loam volcanic soil (VS), and clay red soil (RS). Plant physiological and growth parameters, nutritional and nutraceutical profile of seeds, and nutrient bioavailability in the substrates, before and after cultivation, were determined.

**Results:**

Net photosynthesis was lower in plants in pure regolith, while the addition of compost restored assimilation at a similar rate to that of the other substrates. Both regolith-based substrates reduced the biomass accumulation, but seed production improved in R70C30 (+61.9% than R100), giving similar yield compared to VS and S70C30. The chemical fertility and nutrient bioavailability improved after cultivation of the fava bean Fabaceae crop in succession to potato (e.g., in R100, +52% organic C, +19% N, and +27% S). The easily bioavailable nutrients declined over time, while the potentially bioavailable fraction increased, indicating a strengthening interaction with the substrate adsorption surface.

**Discussion:**

The growth on pure regolith simulant MMS-1 reduced the plant growth and seed production; however, the amendment with green compost improved the nutrient bioavailability of MMS-1, with positive effects on the yield, harvest index, and nutritional quality of fava bean seeds, at similar level to volcanic soils.

## 1 Introduction

The long-term manned missions in Space and the permanence of humans in orbital outposts or planetary colonies will require specific technologies to regenerate resources, also exploiting the *in-situ* resource utilization (ISRU). Bioregenerative Life Support Systems (BLSSs) can help to sustain human life in Space by performing several fundamental regenerative functions. BLSSs are artificial ecosystems, including selected organisms, organised in successive steps of recycling, to reconvert the crew wastes (carbon dioxide, faeces and urine, food remains) in oxygen, potable water, and edible biomass (Häder et al., 2018).

Higher plants are effective bioregenerators, as they are able to renew air via photosynthesis, to purify water through transpiration, and to recycle waste through nutrient uptake, also providing fresh food along with physical and psychological health benefits for astronauts (Wheeler, 2010). Several salad and staple crops have been selected for cultivation in BLSSs based on specific criteria, including compact plant size, short growing cycle, high productivity and harvest index (as a ratio of edible part to total biomass), high produce nutrient and nutraceutical value, and low requirement of fertilizers (De Micco et al., 2012).

Fava bean (*Vicia faba* L., botanical family Fabaceae) is a cool-season, annual grain legume, widely cultivated in the temperate zone of the northern hemisphere (Duc et al., 2015). The plant produce is mostly harvested as dry seeds, but fresh seeds or pods can also be consumed (Hans et al., 2022). Faba bean cultivation provides valuable ecological and environmental benefits in sustainable agriculture, also thanks to the aptitude to host pollinating insects and to establish symbiosis with nitrogen-fixing rhizobia bacteria, which reduces the crop requirement of chemical fertilizers (Karkanis et al., 2018). Plant height widely ranges mainly depending on the genotype and cultural conditions. Seed yield varies in the range of 5.0-7.0 t ha^-1^ (fresh seed, average moisture content 84%) or 1.0-3.5 t ha^-1^ (dry seed, average moisture content 11%), depending on the cultivar and pedo-climatic conditions, and the harvest index is 40- 60% (on a dry matter basis), and the duration of the growing cycle widely varies depending on the time of sowing and the temperatures, from lasts 4 months in spring and to 7 months in fall-winter (Bangar and Kajla, 2022). Seeds show the following basic compounds composition (as dry matter percentage): proteins 17.6-34.5%, total starch 33.2-53.4%, and lipids 0.52-2.80%, with similar variations in European and Asian seed collections (Chaudhary and Kajla, 2022 Trypsin inhibitor activity in faba bean seeds is generally lower compared to soybean. In some accessions (usually white-flower genotypes) seeds are tannin-free, whereas in others (coloured-flower genotypes) condensed tannins are concentrated in the seed coat. The mean content of vicine and convicine, glycosides with anti-nutritional properties responsible for the risk of favism in humans, is 6–12 g/kg DM in conventional genotypes and approximately 0.5 g/kg DM in vc- homozygotes, concentrated in cotyledons (Bangar and Kajla, 2022).

Based on the above-mentioned criteria, fava bean would be a good candidate for space cultivation, as plants can have a compact size and high productive performance, and seeds are a good source of proteins, carbohydrates, B-group vitamins, and minerals (Crépon et al., 2010). However, it is a typical open-field crop, hence most the data about plant performance comes from studies on soil in outdoor conditions, and only a few reports concern soilless cultivation in greenhouse (Alza et al., 2024) or controlled environment (Vestland, 2024).

Faba bean plants grow better in fine-textured soil but can be successfully cultivated in a wide range of soil types, when properly irrigated and fertilised. The optimal soil pH is around 7.0. Plants adapt well to cold climates while are sensitive to heat and drought, and the optimal temperature is 20 °C (Bangar and Kajla, 2022), and can exhibit different photoperiodic response, being classified as either day-neutral or long-day (critical photoperiod for flowering 9.50-12.00 hours), depending on the genotype (Patrick and Stoddard, 2010).

Substrate is a crucial aspect for space farming, where fertile soils are not available and local materials need to be adapted as growing media. Consequently, in the view of exploration missions of Moon and Mars, the growth on native regoliths of the candidate crops, belonging to different plant types (leafy and fruit vegetables, tuber plants), must be tested. The use of regolith simulants amended with organic matrixes, as well as their contribution to the disposal of organic streams in space colonies, has been scarcely investigated (Gilrain et al., 1999; Mortley et al., 2000; Wamelink et al., 2014). Within research projects on plant cultivation in BLSSs funded by the Italian Space Agency (ASI), we studied the use of Martian and Lunar regolith simulants, also in mixture with vegetal or animal organic matter (from green compost and horse/swine manure, respectively), in terms of both substrate characterization and crop performance, in model-species of leaf vegetables (i.e., lettuce; Caporale et al., 2022, Caporale et al., 2023a; Duri et al., 2020, Duri et al., 2022a, Duri et al., 2022b), seed crops (i.e., soybean; Caporale et al., 2023a), and tuber crops (i.e., potato; Caporale et al., 2023b; Caporale et al., 2024). Results revealed that both pure regoliths are unsuitable to sustain the plant growth, even in the presence of fertigation; however, the organic amendment improved physicochemical and hydraulic features and slightly reduced the alkaline pH, allowing a thriving plant growth of these crops.

The MMS-1 is a coarse, alkaline material (pH 8.86), consisting of a mixture of plagioclases, amorphous components, and zeolite, containing some essential plant nutrients (e.g., K, Ca, Mg, and Fe), but lacking organic C, N, P, fine particles with colloidal properties and a suitable water retention (Caporale et al., 2020). After a successful greenhouse tuber-to-tuber cultivation in pot of potato on Mars MMS-1 alone and in mixture with green compost (30% in volume), in a first experiment with soybean (*Glycine max* L. Merr.) in the same substrates, serious symptoms of wilting, leaf yellowing and falling occurred in the stages of seed germination and seedlings establishment. This response, presumably due to multiple reasons (i.e., poor water retention, alkaline pH, low bioavailability of nutrients and high concentration of Na), confirmed the limits of Martian regoliths, and highlighted a great sensitivity of soybean compared to other corps previously tested. Hence, we monitored the plant adaptation during the first stages of development in pure MMS-1 (R100) with mixtures of an acid blond sphagnum peat at 85:15 (R85P15) and 70:30 (R70P30) in volume, in soybean plants directly sown on the substrates or transplanted after sowing in peat, in pot under laboratory conditions (Caporale et al., 2023a). We used peat to exploit its acidic reaction, even though it could not be an amendment for space farming. The acidity of peat (4.34) mitigated the alkalinity of MMS-1 (pH 8.86), reducing pH to 6.98 (R85P15), or 6.33 (R70P30). Seed germination reached the highest percentage in the shortest time in R85P15. A significant interaction Substrate x Planting procedure was found in several parameters, with the best performance in plants from direct seed sowing on both regolith mixtures with peat.

To develop efficient and long-lasting cultivation systems, it is therefore crucial to study the property dynamics of the Martian regolith simulant-based substrates over an appropriate crop succession, for instance, with a soil-improving crop (i.e., nitrogen-fixing Leguminosae) after a nutrient-depleting, exploiter crop (i.e., potato). In this regard, by the current experiment, we assessed the fava bean plant growth performance on the MMS-1 simulant, pure and amended with a commercial green compost (70:30 v:v), compared to a pure and amended fluvial sand, a volcanic sandy-loam soil and a red clay soil. Each growth substrate was the same where potato plants were grown in the previous experiment (Caporale et al., 2023b; Caporale et al., 2024). Particularly, we evaluated the gas exchange, plant growth parameters, the ability to complete the seed-to-seed cycle in fava bean plants grown in pot in an unheated glasshouse, in Mediterranean climate, and the nutritional quality of fava bean seeds. Moreover, we monitored the evolution of the chemical properties and nutrient bioavailability in the substrates over time, as affected by crop succession (fava bean post potato plants). To the best of our knowledge, none of the previous space-oriented studies on plant growth on Lunar or Martian regolith simulants dealt with the evolution over time of chemical properties of the pure substrates or their mixture, also considering the effects of crops with different behaviour in terms of effects on soil fertility.

## 2 Materials and methods

The experiment was carried out at the experimental facilities of the Department of Agricultural Sciences of the University of Naples Federico II in Portici (Naples, Italy, 40°49' N, 14°20' E), from December 28, 2022, to April 13, 2023. The fava bean cultivar 'Sfardella' (Semi Orto Sementi s.r.l., Sarno, Salerno, Italy) was selected as suitable for the test according to the main criteria for cultivation in BLSSs: medium plant size, vigorous plants adaptable to the most diverse climatic conditions, medium cycle, high seed productivity, very tender grain with a particularly sweet flavour. Seeds were disinfected with a 70% ethanol solution, rinsed with deionized water and placed in aluminium trays, between 2 layers of cotton wool soaked in deionized water, and stored in growth chamber in the dark (temperature 12.5°C, relative humidity 70%), for 14 days from December 28, 2022. In total, 90 seeds were divided into 3 trays (30 seeds/tray). The germination rate was 90%.

Seedlings were transplanted in unheated glasshouse on January 11, 2023, in black plastic pots (22 cm diameter) containing 4 L of substrate. Six substrates were compared: pure Mojave Mars regolith simulant MMS-1 (R100), MMS-1 amended with green compost 70:30 v:v (R70C30), a pure fluvial quartz sand (S100), sand mixed with green compost 70:30 v:v (S70C30), a sandy-loam volcanic soil from Campania region (Italy) (VS), and a clay red soil from Sicily region (Italy) (RS). In total, 5 plastic pots, each containing 2 plants, were prepared (10 plants per substrate), and 5 plants were considered as replicates per each treatment.The green compost (Vivai Gardea, Villafranca di Verona, Italy), from the natural, slow microbial decomposition of pruning waste and grass cuttings (3 months in controlled conditions), showed a low C/N ratio, implying a good N availability, and a good aromatic moiety, guaranteeing the stability of the organic matter and a gradual mineral release over time (Caporale et al., 2020). However, the high pH (8.25) did not allow to reduce the pH of the mixture R70C30 to the sub-neutral level required to ensure the proper nutrient availability (Caporale et al., 2020).

Plants were fertigated starting 14 days after transplanting (DAT), using the Hoagland half strength nutrient solution, modified by Wheeler et al. (2008), with EC 2.0 dS m^-1^ and pH 5.8. The frequency and volume of pulses were established based on the water potential of the substrates, measured with tensiometers (Jet-filled Model 2725ARL, length 18", Soilmoisture Equipment Corp., CA), positioned in the different substrates, and the number of interventions per week varied from 3 (vegetative phase) to 7 (flowering and pod filling), alternating 2 fertigations with one irrigation with only water, to prevent the accumulation of salts in the substrate.

Air temperature and relative humidity in the glasshouse were recorded every 10 minutes with a RoHS wireless Bluetooth data logger. During the plant vegetative growth and flowering (up to pod appearance), the average day/night temperatures were 20.3 ± 7.7/10.3 ± 3.4°C, and the relative humidity 58.5 ± 17.3%/69.2 ± 12.3%, while in the stage of pod filling, they were 24.2 ± 8.4/13.0 ± 2.5°C and 51.7 ± 18.1%/66.5 ± 11.2%, respectively (Mean value ± Standard Deviation). The natural day length increased from 9 hours and 31 minutes to 11 hours and 54 minutes in vegetative plant growth and flowering (January 11th to March 14th, 2023); and from 11 hours and 56 minutes to 13 hours and 15 minutes during the pod filling stage (March 15th to April 13th, 2023).

### 2.1 Chemical characterization of cultivation substrates

At the fava bean plant sampling (92 DAT), the root biomass was separated from the growing substrates. The soil adhering to the roots (rhizo soil, RH) was separated from the bulk soil (BK). The RH and BK soil samples were air-dried and sieved at 2 mm for the chemical analyses.

The concentrations of organic C, total N and S were determined by a Micro Elemental Analyser (UNICUBE^®^, Elementar, Hesse, Germany), using sulphanilamide (Elementar, 99.5%) as calibration standard. A separate measure of carbonates was performed to determine the organic C from the total C content.

The easily and potentially bioavailable fractions of the nutrients were extracted from the substrates, after fava bean cultivation cycle, by 1 M NH4NO3 (solid/solution ratio: 1/25; reaction time: 2 h) and 0.05 M EDTA at pH 7 (solid/solution ratio: 1/10; reaction time: 1 h), respectively. The extracts were filtered and analyzed by Inductively Coupled Plasma–Mass Spectrometry (ICP-MS, Thermo Scientific iCAP Q, Waltham, MA, USA). The certified reference material BCR 700 was also analyzed for quality check of EDTA extractions (recovery at ±12% of the certified values).

The pH in the growing media was measured by Hanna Instruments 210 pH meter in ultrapure water (solid/solution ratio: 1:2.5), while the electrical conductivity (EC) by COND 70 + XS conductivity meter (solid/solution ratio: 1:5).

### 2.2 Plant growth and seed yield

The plant growth, in terms of number of stems, leaves and pods, and plant height was monitored once a week in 5 plants per treatment. At the harvest (92 DAT), plant leaf area was measured through a non-destructive analysis of digital images of all the plant leaves, using the ImageJ software 1.53 k (Wayne Rasband Nat. Inst. of Health, USA), and the fresh weight of stems, leaves, pod valves, seeds, and roots, and the dry biomass of each portion, after oven drying at 80 °C, were determined with an analytical balance (XS Mod. Bl 2002 Basic, XS Instruments, Italy), in 5 plants per treatment.

### 2.3 Gas exchange and chlorophyll a fluorescence measurements

Leaf gas exchange parameters, including net CO_2_ assimilation rate (A), stomatal conductance (gs), and transpiration rate (E), were assessed using a portable infrared gas analyser (ADC LCi, BioScientific Ltd., Hoddesdon, UK) fitted with a broad-leaf chamber (6.25 cm² cuvette area). Measurements were performed on February 15 and March 14 (35 and 62 DAT, respectively, corresponding to vegetative growth and beginning of flowering) on one fully expanded, sunlit leaf, in five plants per treatment (5 leaves in total), under ambient Photosynthetic Photon Flux Density (PPFD).

Chlorophyll fluorescence emission was evaluated on the same leaves, using a portable fluorimeter kit (Plant Stress Kit, Opti-Sciences, Hudson, NY, USA) equipped with a light sensor. The ground fluorescence (Fo) was induced by a low-intensity blue LED light (~1-2 μmol m^-^² s^-^¹), after 30 minutes of dark adaptation. The maximal fluorescence in the dark (Fm) was triggered with a saturating light pulse (3000 μmol m^-^² s^-^¹ for 1 second). The maximum quantum efficiency of PSII photochemistry (Fv/Fm) was calculated as (Fm−Fo)/Fm, following the formula proposed by Kitajima and Butler (1975). Fluorescence measurements in the light were taken between 10:00 and 12:00 am under ambient PPFD, ranging from 495 to 600 μmol m^-^² s^-^¹. The PSII quantum yield of electron transport (ΦPSII) was calculated as described by Genty et al. (1989), while non-photochemical quenching (NPQ) was determined using the method of Bilger and Björkman (1990).

### 2.4 Photosynthetic pigment content determination

Photosynthetic pigments in terms of chlorophyll a, chlorophyll b, total chlorophylls, and carotenoids were extracted from one fully developed leaf per 5 plants per treatment, during plant vegetative phase. The extraction was performed by grinding the samples in ice-cold 100% acetone, which were then centrifuged at 5000 rpm for 5 minutes in a D2012 Scilogex microcentrifuge (Sinergica Soluzioni, Milan, Italy). The supernatants were collected, and photosynthetic pigments were quantified with a spectrophotometer Onda Touch UV-21 (Onda, Shanghai, China) at the wavelengths of 470, 645, and 662 nm. The pigment content was calculated following the formula of Lichtenthaler (1987) and expressed in µg cm^-2^.

### 2.5 Seed quality

The elemental profile in the fava bean seeds was assessed as follows: i) the C, N, and S concentrations were measured by the Micro Elemental Analyser UNICUBE^®^ (Elementar, Hesse, Germany) in 2 mg of dried biomass; the concentrations of K, P, Mg, Ca, Na, Fe, Zn, B, Mn, and Cu were analyzed by ICP-MS (Thermo Scientific iCAP Q, Waltham, MA, USA), after a microwave-assisted digestion of 500 mg dried sample (Milestone Start D, Sorisole, BG, Italy) with HNO3 65% and HCl 37%.

To assess the polyphenol profile, freeze-dried samples were extracted following the method reported by Doppler et al. (2022), with slight modifications. Briefly, 0.2 g of each sample was weighed, and 2 mL of 0.1% acidified MeOH/water solution (70:30 v/v) was added. After vortexing for 1 minute, the samples were sonicated for 15 minutes and centrifuged at 14,800 rpm and 4°C for 10 minutes. The supernatants were then collected, filtered through 0.45 µm polytetrafluoroethylene (PTFE) filters, and diluted prior to polyphenol identification by high-performance liquid chromatography (HPLC). The phenolic characterization was performed through a chromatographic approach as reported recently by Chiacchio et al. (2022). For the analysis, an HPLC SHIMADZU equipped with UV/VIS SPD-20° detector (Prominence, USA) and a Prodigy ODS3 100 Å column (250 mm × 4.6 mm particle size 5 μm) (Phenomenex. CA. USA) were used. The mobile phases consisted of water at 0.2% formic acid (solvent A) and acetonitrile/methanol (60:40 v/v) (solvent B). The injection volume was 20 μL with a flow rate of 1 mL/min and the gradient was programmed as follows: 20% B (2 min). 30% B (8 min). 40% B (18 min). 50% B (26 min). 90% B (34 min). 90% B (37 min). 20% B (39 min). 20% B (43 min). The phenolic compounds identified were: catechin, p-hydroxybenzoic acid, epicatechin, caffeic acid, p-coumaric acid, rutin, trans-ferulic acid, naringin, and myricetin. To identify the unknown compounds and confirm the acquired identification, spectrometry analysis was also performed by using a Vanquish Core LC system coupled to a high-resolution quadrupole Orbitrap mass spectrometer (Exploris 120, Thermo Fisher Scientific, Waltham, MA). The same chromatographic condition was used for HPLC analysis. The tune method parameters were set as previously shown (Chiacchio et al., 2022). This allowed the identification of apigenin 7-O-glucoside and myricetin 3-O-glucoside whose concentrations were expressed as equivalent of apigenin and myricetin, respectively. All the analyses were conducted in three replicates and the results were expressed as µg/g of dry weight using a calibration curve built with the corresponding standard compound. All the reagents were analytical grade and were purchased from Sigma-Aldrich (Milan, Italy), as were methanol, water, formic acid, acetonitrile, catechin, epicatechin, trans-ferulic acid, rutin, p-coumaric acid, naringin, p-hydroxybenzoic acid, and myricetin. The retention times of the identified molecules are reported in **Supplementary Table S1**.

### 2.6 Statistical analysis of data

Each substrate treatment consisted of 5 pots, distributed randomly on the bench. For growth parameters and seed production, 5 plants were used as replicates and data were analysed by one-way analysis of variance (ANOVA) using the SPSS 28 software package (www.ibm.com/software/analytics/spss), and means were compared by Tukey's multiple-range *post-hoc* test, performed at a significance level of P ≤ 0.05, P ≤ 0.01 and P ≤ 0.001. Data on nutrient bioavailability in the substrates were analysed by two-way ANOVA, with the following sources of variance: six different substrates (S); and two different soil types (RB; i.e., RH vs. BK). Means were compared through Tukey's multiple-range *post-hoc* test, at p < 0.05. To test the influence of the two independent factors, i) Treatment (T), ii) Date (D) and their interaction (T x D) on gas-exchange (NP, gs, E) and photochemical (ΦPSII, ETR, Fv/Fm, NPQ) parameters, a two-way ANOVA was performed using the SPSS 28 software package (www.ibm.com/software/analytics/spss; SPSS, Chicago, IL, USA). When the interactions were significant, a one-way ANOVA was performed, separating the values according to Tukey test with a p value ≤ 0.05. The assumptions of ANOVA were verified prior to the analyses: normality of residuals was tested using the Shapiro-Wilk test and homogeneity of variances was checked with Levene's test.

## 3 Results

### 3.1 Plant physiology

**Figure 1:**
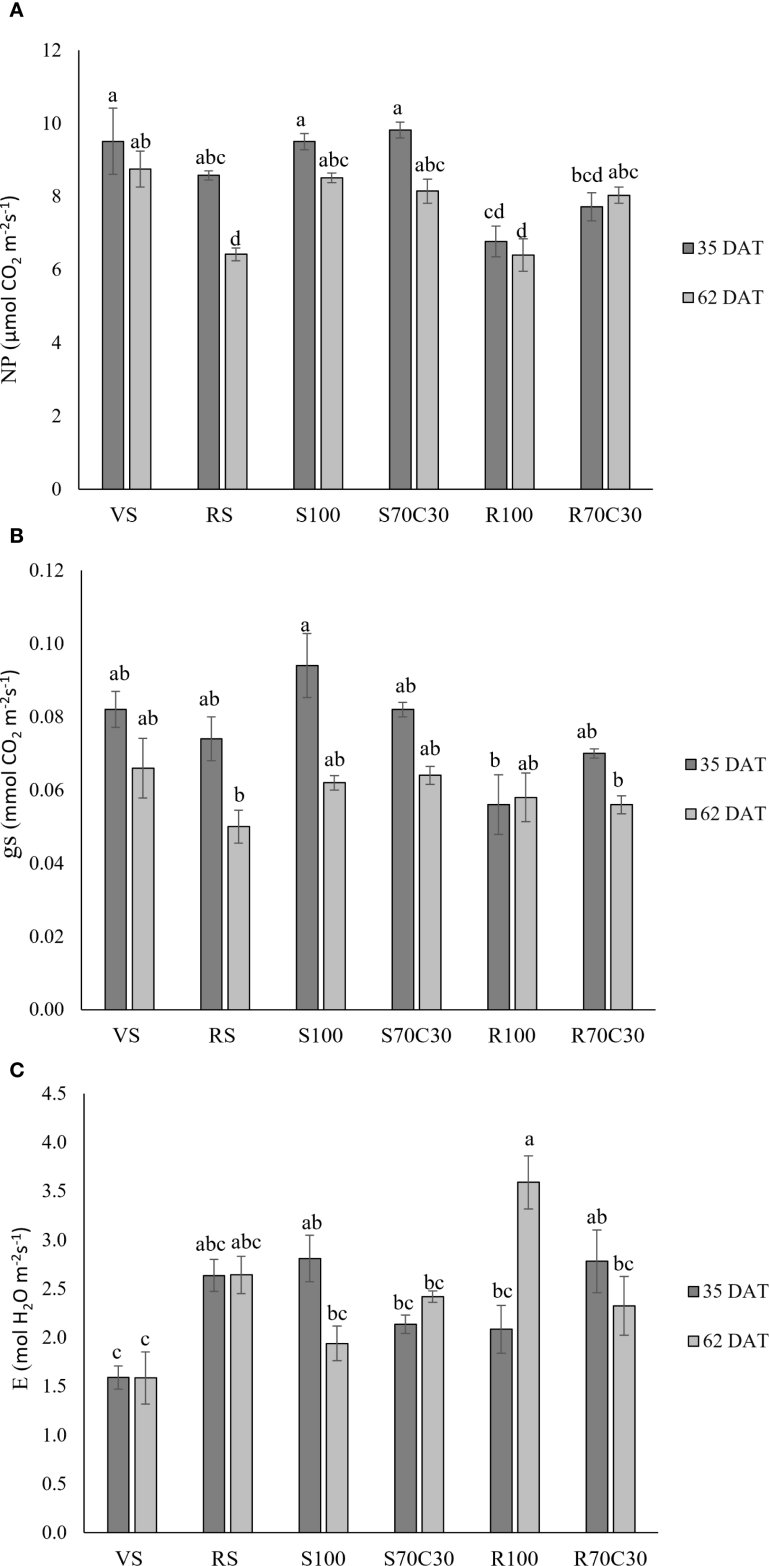
**(A)** Net-photosynthesis (NP), **(B)** transpiration rate (E), and **(C)** stomatal conductance (gs) in fava bean cv. 'Sfardella' plants grown on the six different substrates: i) volcanic soil (VS), ii) red clay soil (RS), iii) fluvial quartz sand (S100), iv) fluvial quartz sand mixed with green compost (S70C30), v) Mojave Mars regolith Simulant MMS-1, (R100), and vi) Mojave Mars regolith Simulant mixed with green compost (R70C30). Mean value ± Standard error, n = 5 measured at 35 and 62DAT. Different letters indicate significant differences among the treatments at p ≤ 0.05.

**Figure 1** shows the gas-exchange parameters measured in fully developed leaves of fava bean at 35 and 62 days after transplanting (DAT), corresponding to the vegetative growth stage and the beginning of flowering, respectively. Net photosynthesis (NP, **Figure 1a**; **Supplementary Table S2**) was significantly affected by both treatment (p ≤ 0.001) and date (p ≤0.001), with a significant interaction (T × D, p ≤ 0.05). NP showed the highest values in VS, S100, and S70C30 at 35 DAT, whereas the lowest values were observed in RS and R100 at 62 DAT. Stomatal conductance (gs, **Figure 1b**; **Supplementary Table S2**) was strongly influenced by date (p ≤ 0.001) but not by treatment (ns), although the interaction (T× D) was significant (p ≤ 0.05). The highest gs values occurred in S100 at 35 DAT, while the lowest were recorded in RS and R70C30 at 62 DAT and in R100 at 35 DAT. All other treatments showed intermediate values. For transpiration rate (E, **Figure 1c**; **Supplementary Table S2**), treatment (p ≤ 0.001) and the interaction (p ≤ 0.001) were significant, whereas date alone was not (ns). The highest transpiration was recorded in R100 at 62 DAT, while VS at both 35 and 62 DAT showed the lowest values.

**Figure 2:**
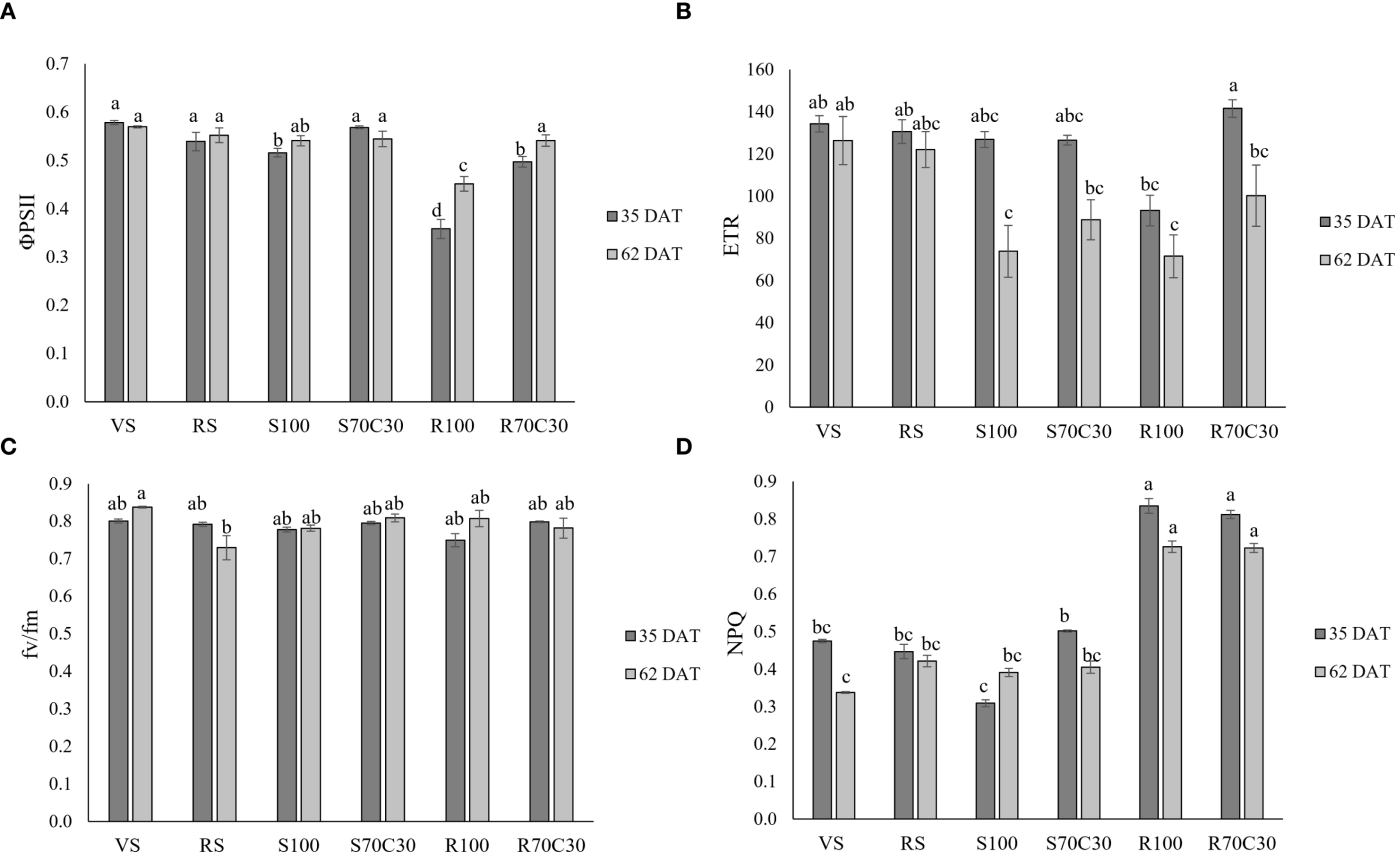
**(A)** The PSII quantum yield of linear electron transport (φPSII), **(B)** electron transport rate (ETR), **(C)** The maximum quantum efficiency of PSII photochemistry (fv/fm), and **(D)** the non-photochemical quenching (NPQ) in fava bean cv. 'Sfardella' plants grown on the six different substrates: i) volcanic soil (VS), ii) red clay soil (RS), iii) fluvial quartz sand (S100), iv) fluvial quartz sand mixed with green compost (S70C30), v) Mojave Mars regolith Simulant MMS-1, (R100), and vi) Mojave Mars regolith Simulant mixed with green compost (R70C30). Mean value ± Standard error, n = 5 measured at 35 and 62 DAT. Different letters indicate significant differences among the treatments at p ≤ 0.05.

**Figure 2** reports the indexes of leaf photochemical efficiency measured at the same dates. The quantum yield of PSII linear electron transport (ΦPSII, **Figure 2a**; **Supplementary Table S2**) was significantly affected by both treatment (p ≤ 0.001), date (p ≤ 0.01), and their interaction (p ≤ 0.01). At both sampling dates, ΦPSII was higher in VS, RS, S70C30, and lower in R100. In R70C30, values increased at 62 DAT compared to 35 DAT. Overall, the lowest values were found in R100 at 35DAT. The electron transport rate (ETR, **Figure 2b**; **Supplementary Table S2**) was significantly affected by treatment (p ≤ 0.001), date (p ≤ 0.001), and their interaction (p ≤ 0.05). R70C30 showed the highest ETR at 35 DAT, while R100 and S100 maintained significantly lower values at 62 DAT. The maximum quantum efficiency of PSII photochemistry (fv/fm, **Figure 2c**; **Supplementary Table S2**), varied less across treatments, with significant effects of treatment (p ≤ 0.01) and T × D interaction (p ≤ 0.01). VS at 62 DAT showed the highest fv/fm, while RS at 62 DAT presented the lowest values. Finally, the non-photochemical quenching (NPQ, **Figure 2d**; **Supplementary Table S2**) revealed a clear trend of enhanced thermal dissipation in the regolith-based substrates (R100 and R70C30), which were significantly higher than all other treatments (p ≤ 0.001) at both sampling dates. In contrast, VS at 62 DAT and S100 at 35 DAT showed the lowest NPQ, confirming reduced engagement of non-photochemical energy dissipation in sand-grown plants.

**Figure 3:**
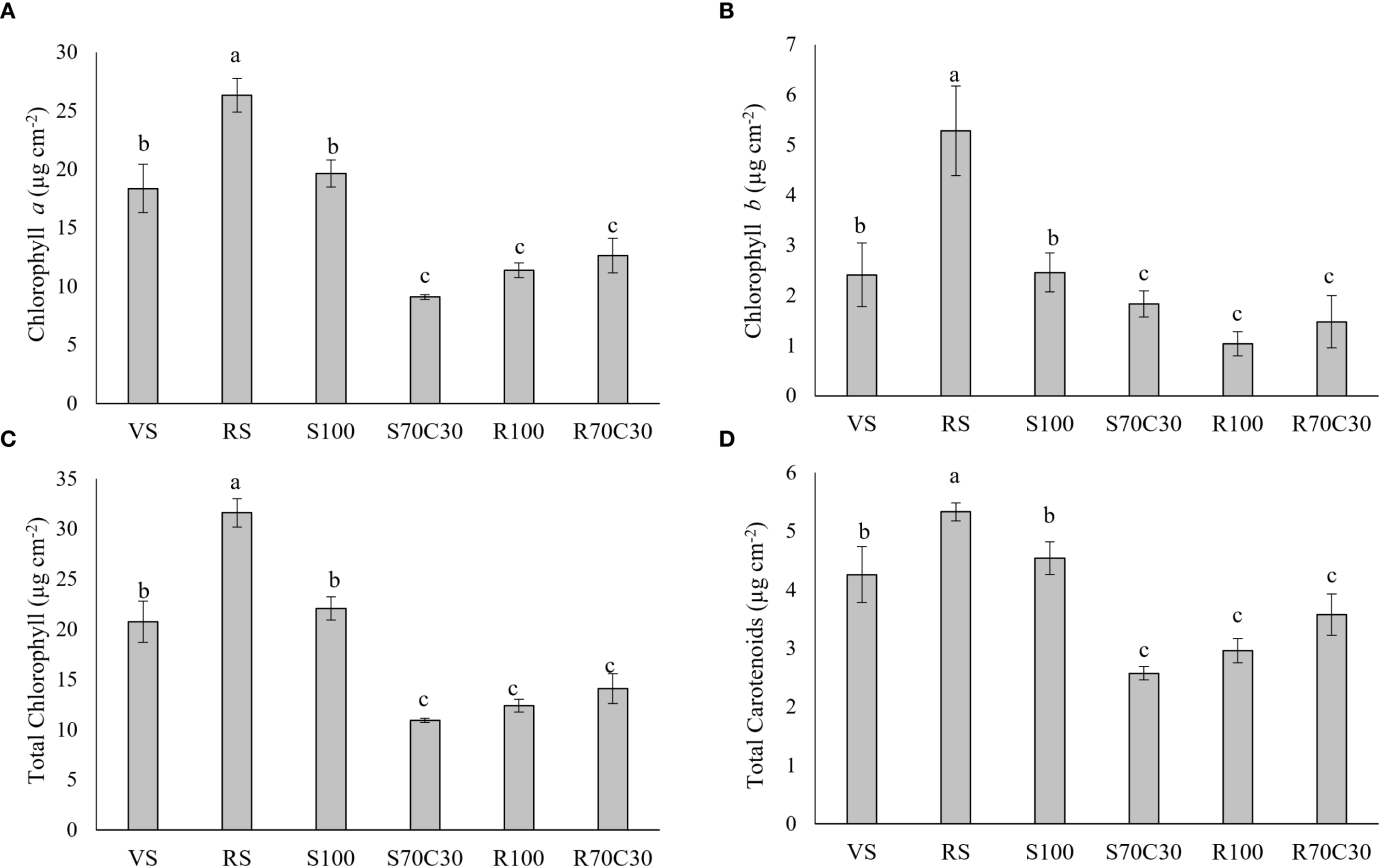
**(A)** Chlorophyll a, **(B)** chlorophyll b, **(C)** total chlorophylls, and **(D)** total carotenoids in fava bean cv. 'Sfardella' plants grown on the six different substrates: i) volcanic soil (VS), ii) red clay soil (RS), iii) fluvial quartz sand (S100), iv) fluvial quartz sand mixed with green compost (S70C30), v) Mojave Mars regolith Simulant MMS-1, (R100), and vi) Mojave Mars regolith Simulant mixed with green compost (R70C30). Mean value ± Standard error, n = 5 measured at 35 and 62DAT. Different letters indicate significant differences among the treatments at p ≤ 0.05.

All photosynthetic pigments of fava bean leaves (**Figure 3**) showed the same trend of variation among the different substrates, with the highest content of both chlorophylls and carotenoids in RS, followed by VS and S100, and the lowest in S70C30, R100, and R70C30.

### 3.2 Plant growth

**Tables 1** and **2** show the main growth parameters recorded in fully developed plants of fava bean grown on the tested substrates. Plant height was similar in all substrates except in red soil (RS), which gave significantly shorter plants. However, the overall plant growth did not reflect the plant height, and plants on volcanic soil (VS) and sand and compost mixture (S70C30) produced the largest leaf area (**Table 1**) and the greatest total aerial biomass (**Table 2**). The growth on pure Mars regolith simulant MMS-1 (R100) reduced the aerial biomass accumulation compared to VS and S70C30 to similar values to the other substrates (**Table 2**); however the addition of green compost (R70C30) seemed to increase the number of leaves and pods, and the plant leaf area, even though these effects were not statistically significant (**Table 1**), and strongly improved the seed production (+61.9% with respect to R100), giving the same productive performance of VS and S70C30 (**Table 2**). In pure regolith (R100), the number of seeds was similar to VS and S70C30, nevertheless, since they were very small, the total yield was among the lowest values (**Table 2**).

**Table 1:** Main growth parameters of fully developed plants of faba bean cv. 'Sfardella' grown on volcanic sandy-loam soil (VS), red clay soil (RS), pure fluvial sand (S100), sand mixed with green compost (70:30 v:v; S70C30), pure MMS-1 simulant (R100), MMS-1 amended with green compost (70:30 v:v; R70C30).

Substrates	Plant height (cm)	Number of leaves (no. plant^-^¹)	Plant leaf area (cm^2^ plant^-1^)	Number of pods (no. plant^-^¹)	Number of seeds (no. plant^-^¹)
VS	36.75 ± 1.22 a	78.90 ± 3.18 a	746.8 ± 21.0 a	4.30 ± 0.41 a	8.10 ± 0.29 ab
RS	28.00 ± 0.75 b	67.80 ± 2.92 ab	358.0 ± 33.3 b	2.80 ± 0.6 ab	5.70 ± 0.51 b
S100	34.75 ± 2.04 a	54.60 ± 4.35 ab	368.5 ± 34.3 b	2.50 ± 0.16 b	5.60 ± 0.24 b
S70C30	33.30 ± 0.41 a	73.70 ± 3.56 ab	614.1 ± 37.3 a	3.60 ± 0.4 ab	8.50 ± 0.63 a
R100	36.05 ± 1.17 a	49.00 ± 7.54 b	349.9 ± 64.5 b	2.50 ± 0.32 b	7.10 ± 1.08 ab
R70C30	33.25 ± 0.69 a	69.30 ± 9.37 ab	432.3 ± 13.8 b	3.00 ± 0.32 ab	6.90 ± 0.37 ab
*Significance*	***	**	***	*	**

106 DAT; Mean values ± Standard Errors; n=5. Different letters within each column indicate significant differences according to one-way ANOVA, Tukey's multiple-range test (*p<0.05; **p<0.01; ***p<0.001).

**Table 2:** Seed yield, total fresh and dry mass, and dry weight (DW) of the main parts of the plant of faba bean cv. 'Sfardella' grown on volcanic sandy-loam soil (VS), red clay soil (RS), fluvial sand, alone (S100) and in mixture with a commercial green compost (70:30 v:v; S70C30), and Mojave Mars regolith Simulant MMS-1, alone (R100) and in mixture with a commercial green compost (70:30 v:v; R70C30), at the harvest (106 DAT).

Substrates	Seed production (g FW seeds plant^-1^)	Total aerial biomass (g FW plant^-1^)	Total dry biomass (g plant^-1^)	DW seeds (g plant^-1^)	DW pods valves (g plant^-1^)	DW leaves (g plant^-1^)	DW stems (g plant^-1^)	DW roots (g plant^-1^)	D.m. % seeds (% of fresh weight)
VS	19.48 ± 0.55 a	90.81 ± 2.57 a	18.71 ± 0.70 a	4.34 ± 0.17 a	4.64 ± 0.22 a	4.20 ± 0.26 a	3.54 ± 0.14 a	1.98 ± 0.16	22.29 ± 0.60
RS	12.25 ± 1.14 b	55.91 ± 3.10 c	11.42 ± 0.55 b	2.59 ± 0.25 b	2.96 ± 0.23 b	1.84 ± 0.10 b	2.16 ± 0.07 c	1.86 ± 0.11	21.14 ± 0.19
S100	13.65 ± 0.92 b	59.48 ± 2.65 bc	12.68 ± 0.58 b	2.96 ± 0.20 b	3.03 ± 0.12 b	1.93 ± 0.20 b	2.76 ± 0.14 bc	2.00 ± 0.23	21.68 ± 0.33
S70C30	21.82 ± 0.99 a	91.51 ± 3.07 a	18.54 ± 0.58 a	4.96 ± 0.31 a	4.50 ± 0.30 a	3.66 ± 0.33 a	3.11 ± 0.09 ab	2.32 ± 0.28	22.64 ± 0.47
R100	11.94 ± 1.09 b	60.24 ± 6.09 bc	12.40 ± 1.55 b	2.40 ± 0.18 b	2.90 ± 0.31 b	1.87 ± 0.42 b	2.66 ± 0.21 bc	2.57 ± 0.58	20.29 ± 0.69
R70C30	19.33 ± 0.46 a	72.42 ± 1.78 b	14.89 ± 0.50 b	4.45 ± 0.31 a	3.29 ± 0.17 b	2.20 ± 0.09 b	2.77 ± 0.15 bc	2.18 ± 0.24	23.13 ± 1.83
*Significance*	***	***	***	***	***	***	***	ns	ns

Mean values ± Standard Errors; n=5. Different letters within each column indicate significant differences according to one-way ANOVA, Tukey's multiple-range test (***p<0.001; ns, not significant).

**Figure 4:**
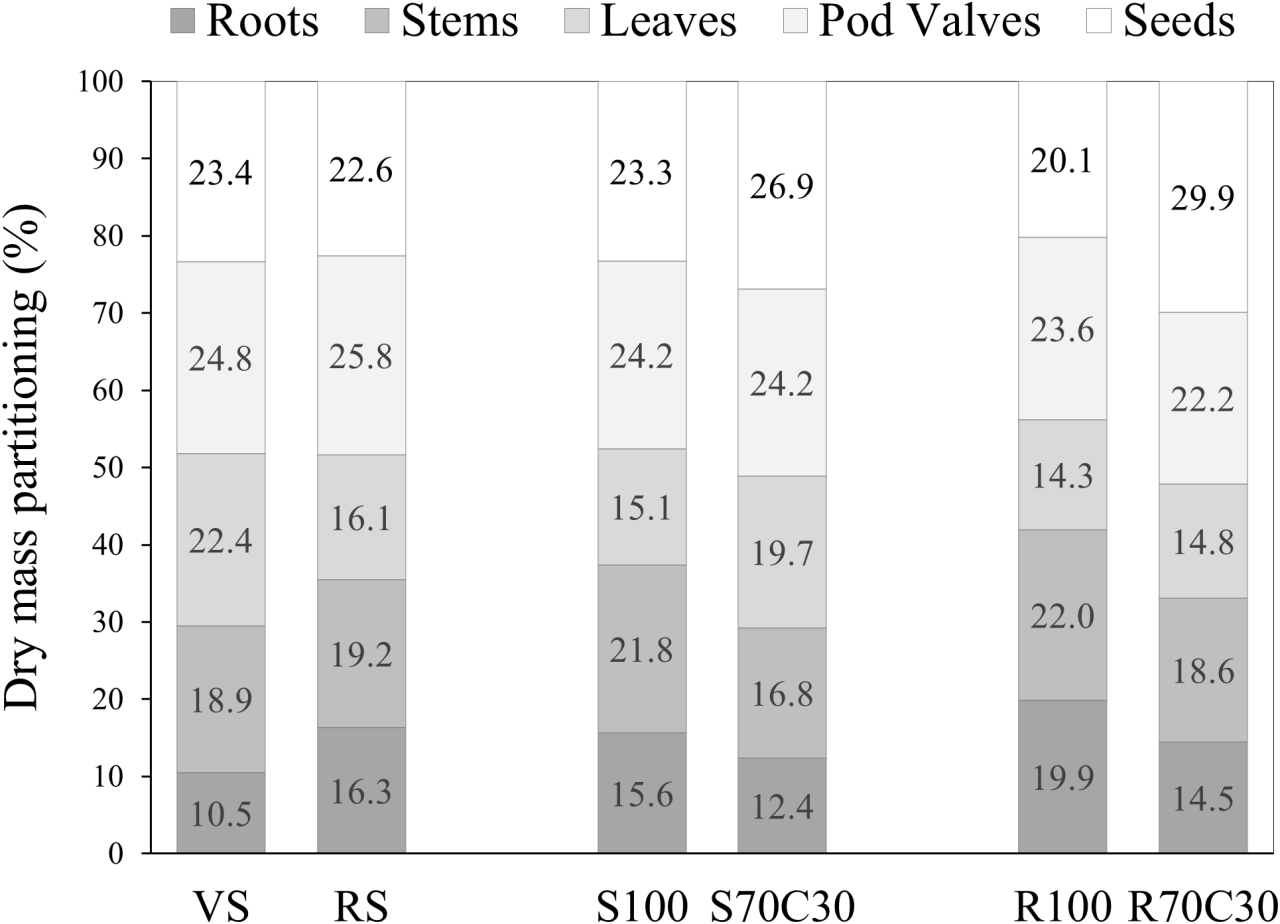
Total dry mass partitioning in the plant organs in faba bean cv. 'Sfardella' grown on volcanic sandy-loam soil (VS), red clay soil (RS), pure fluvial sand (S100), sand mixed with green compost (70:30 v:v; S70C30), pure MMS-1 simulant (R100), MMS-1 amended with green compost (70:30 v:v; R70C30).

Data in **Table 2** report the impact of substrates on the dry weight (DW) of the different plant organs, seeds and stems, leaves, pod valves, and roots (representing cultivation residues), and **Figure 4** shows the dry mass partitioning in these organs as a percentage of the total dry mass. Results revealed that both the mixtures with compost (S70C30 and R70C30) gave the highest harvest index, as incidence of seed dry weight on the total dry weight (29.9% and 26.9%, respectively) and, consequently, the lowest amount of waste (73.1% and 70.1%, respectively). Despite the similar seed yield, VS produced more inedible biomass (76.6% on a dry matter basis) than the compost-based mixtures. No substrate influenced the dry matter percentage of seeds, which was 21.86% on the average of the tested treatments (**Table 2**).

### 3.3 Seed nutritional and nutraceutical quality

The mineral profile of fava bean seeds grown in the various experimental substrates is reported in **Supplementary Table S3** (nutrient concentrations) and **Table 3** (nutrient content, obtained multiplying nutrient concentrations by dry weight of the seeds). Apart from the highly-concentrated C (i.e., the major element of all the biomolecules), fava bean seeds contain great concentrations of N (min-max, 30–41 g kg−1 DW) and K (12–16 g kg−1 DW); they are also a good source of S (2.5-4.2 g kg−1 DW), P (1.8-2.3 g kg−1 DW), Mg (0.9-1.2 g kg−1 DW), Ca (0.7-0.9 g kg−1 DW), and other micronutrients such as Na, Fe, Zn, B, Mn, and Cu (in concentrations <0.1 g kg−1 DW; **Supplementary Table S3**).

**Table 3:** Contents (g plant^-1^, mg plant^-1^ and µg plant^-1^ DW) of main nutrients in grains of fava bean cv. 'Sfardella', grown on volcanic soil (VS), red soil (RS), pure fluvial sand (S100), sand mixed with green compost (70:30 v:v; S70C30), pure MMS-1 simulant (R100), MMS-1 amended with green compost (70:30 v:v; R70C30).

Nutrients	VS	RS	S100	S70c30	R100	R70c30	Sig.
g plant^-1^ DW							
C	1.8 ± 0.1 b	1.0 ± 0.1 c	1.2 ± 0.1 c	2.2 ± 0.1 a	1.0 ± 0.1 c	1.8 ± 0.1 b	***
mg plant^-1^ DW							
N	179 ± 4 a	102 ± 2 c	89 ± 2 cd	173 ± 4 a	79 ± 2 d	143 ± 3 b	***
K	55 ± 1 b	31 ± 1 d	39 ± 1 c	71 ± 2 a	38 ± 1 c	53 ± 1 b	***
S	18 ± 1 a	9.3 ± 0.2 d	8.7 ± 0.2 d	14 ± 0.3 b	6.4 ± 0.1 e	11 ± 0.2 c	***
P	9.3 ± 0.2 b	5.9 ± 0.1 d	5.4 ± 0.1 de	11 ± 0.2 a	4.8 ± 0.1 e	8.0 ± 0.2 c	**
Mg	4.4 ± 0.1 b	2.4 ± 0.1 d	2.9 ± 0.1 c	5.7 ± 0.1 a	2.7 ± 0.1 cd	4.1 ± 0.1 b	***
Ca	3.2 ± 0.1 b	2.4 ± 0.1 cd	2.5 ± 0.1 c	3.7 ± 0.1 a	2.1 ± 0.1 d	2.9 ± 0.1 b	***
µg plant^-1^ DW							
Na	396 ± 9 a	203 ± 5 d	155 ± 3 e	246 ± 6 c	209 ± 5 d	315 ± 7 b	***
Fe	217 ± 5 a	90 ± 2 d	135 ± 3 c	223 ± 5 a	142 ± 3 bc	163 ± 4 b	***
Zn	122 ± 3 a	61 ± 1 c	46 ± 1 d	113 ± 3 a	51 ± 1 cd	90 ± 2 b	***
B	43 ± 1 b	24 ± 1 c	26 ± 1 c	43 ± 1 b	72 ± 2 a	48 ± 1 b	***
Mn	36 ± 1 ab	27 ± 1 c	27 ± 1 c	38 ± 1 a	24 ± 1 c	32 ± 1 b	***
Cu	46 ± 1 a	22 ± 1 cd	23 ± 1 c	32 ± 1 b	18 ± 1 e	19 ± 1 de	***

Mean values ± Standard Errors; n=5. Different letters within each row indicate significant differences according to one-way ANOVA. Tukey's HSD *post hoc* test (*p<0.05; **p<0.01; ***p<0.001; ns, not significant).

The diverse plant growing media produced statistically significant differences in the mineral concentrations in the dry seed biomass, except for C (which is basically taken from atmospheric CO_2_); it is interesting to note that the seeds of plants grown in R100 showed a similar nutritional quality to seeds produced in terrestrial soils and compost-amended substrates, which was significantly better than that of seeds from S100 (**Supplementary Table S3**). These results are related to the nutrient content or bioavailability in the substrates (**Tables 4**, **5**) and to the seed biomass collected from the different growing media (**Table 2**). This implies that R100 treatment produced a scarce seed yield but of a high nutritional quality; compost supply allowed fava bean plants to raise their seed dry biomass and to maintain a high qualitative standard, in line with the findings observed in the two terrestrial soils (VS > RS).

**Table 4:** Concentrations (mg kg^-1^ DW) of main macro and micronutrients extracted by 1M NH_4_NO_3_ from sandy-loam volcanic soil (VS), clay red soil (RS), pure fluvial sand (S100), sand mixed with green compost (70:30 v:v; S70C30), pure MMS-1 simulant (R100), MMS-1 amended with green compost (70:30 v:v; R70C30), separated in rhizo (RH) and bulk (BK) soils after fava bean plant growth.

Source of variance	Ca	K	Mg	Na	P	Fe	Mn	Cu	Zn	B
mg kg^-1^ DW										
VS	2799 ± 67 b	1120 ± 61 a	183 ± 12 c	115 ± 5 b	4.4 ± 0.3 c	1.4 ± 0.1 b	0.55 ± 0.14	0.35 ± 0.03 a	0.05 ± 0.01 b	0.26 ± 0.06 c
RS	4640 ± 145 a	253 ± 30 d	228 ± 10 b	69 ± 3 c	0.2 ± 0.1 e	1.4 ± 0.2 b	0.39 ± 0.16	0.05 ± 0.01 d	0.04 ± 0.01 b	0.09 ± 0.01 c
S100	642 ± 12 c	61 ± 3 e	34 ± 1 e	6 ± 1 d	3.5 ± 0.3 cd	0.6 ± 0.1 c	1.14 ± 0.19	0.06 ± 0.01 d	0.03 ± 0.01 c	0.06 ± 0.01 c
S70C30	1099 ± 46 c	260 ± 16 d	133 ± 7 d	18 ± 1 d	23.4 ± 0.8 a	2.2 ± 0.2 a	0.56 ± 0.05	0.19 ± 0.01 b	0.08 ± 0.01 a	0.47 ± 0.03 c
R100	2272 ± 67 b	454 ± 26 c	267 ± 9 b	101 ± 13 b	2.5 ± 0.2 d	0.7 ± 0.1 c	0.87 ± 0.59	0.07 ± 0.01 d	0.03 ± 0.01 c	3.9 ± 0.48 b
R70C30	2772 ± 69 b	899 ± 46 b	361 ± 10 a	195 ± 14 a	15.7 ± 0.6 b	1.0 ± 0.1 bc	0.42 ± 0.06	0.11 ± 0.01 c	0.05 ± 0.01 b	7.7 ± 0.32 a
*Soil (S)*	***	***	***	***	***	***	*ns*	***	***	***
RH	2345 ± 569	471 ± 161	191 ± 46	81 ± 28	8.3 ± 3.9	1.3 ± 0.3	0.83 ± 0.36	0.15 ± 0.06	0.05 ± 0.01	2.0 ± 1.28
BK	2396 ± 620	545 ± 187	211 ± 49	87 ± 32	8.3 ± 3.7	1.2 ± 0.2	0.48 ± 0.14	0.13 ± 0.04	0.05 ± 0.01	2.2 ± 1.37
*RH vs BK (RB)*	*ns*	***	***	*ns*	*ns*	*ns*	*	***	*ns*	*ns*
Soil * RB	*ns*	*	*ns*	*ns*	*ns*	*	*	*	*ns*	*ns*

Mean values ± Standard Errors; n=5. Soil (S), RH vs BK (RB) and their interaction (S x RB) were compared by two-way ANOVA. Tukey's HSD *post hoc* test (* p<0.05; ** p<0.01; *** p<0.001; ns, not significant). Different lowercase letters within each column indicate significant differences (p<0.05).

**Table 5:** Concentrations (mg kg^-1^ DW) of main macro and micronutrients extracted by 0.05M EDTA (buffered at pH 7) from sandy-loam volcanic soil (VS), clay red soil (RS), pure fluvial sand (S100), sand mixed with green compost (70:30 v:v; S70C30), pure MMS-1 simulant (R100), MMS-1 amended with green compost (70:30 v:v; R70C30), separated in rhizo (RH) and bulk (BK) soils after fava bean plant growth.

Source of variance	Ca	K	Mg	Na	P	Fe	Mn	Cu	Zn	B
mg kg^-1^ DW										
VS	14036 ± 212 b	1019 ± 48 a	590 ± 21 b	171 ± 7 b	326 ± 8 c	144 ± 3 d	59.2 ± 1.2 c	23.7 ± 0.5 a	11.8 ± 0.3 a	1.2 ± 0.1 cd
RS	5904 ± 166 d	268 ± 19 e	346 ± 6 d	116 ± 4 c	12.3 ± 1 e	359 ± 20 a	646 ± 8.3 a	14.3 ± 0.2 b	2.4 ± 0.1 d	0.8 ± 0.1 cd
S100	18861 ± 318 a	112 ± 3 f	163 ± 3 e	48 ± 1 d	31.0 ± 2 e	214 ± 4 c	107 ± 2.4 b	0.6 ± 0.1 d	1.2 ± 0.1 e	0.3 ± 0.1 d
S70C30	18838 ± 306 a	349 ± 21 d	375 ± 16 d	67 ± 2 d	215 ± 17 d	318 ± 11 b	105 ± 1.7 b	4.5 ± 0.4 c	6.4 ± 0.4 c	1.5 ± 0.1 c
R100	7142 ± 112 d	448 ± 21 c	471 ± 12 c	176 ± 19 b	392 ± 11 b	11.1 ± 0.4 e	30.1 ± 0.8 c	0.8 ± 0.1 d	2.0 ± 0.2 d	5.8 ± 0.6 b
R70C30	10738 ± 153 c	824 ± 26 b	764 ± 10 a	296 ± 20 a	462 ± 9 a	159 ± 4 d	48.6 ± 0.9 c	4.4 ± 0.2 c	7.2 ± 0.2 b	13.6 ± 0.6 a
*Soil (S)*	***	***	***	***	***	***	***	***	***	***
RH	12609 ± 2336	472 ± 135	439 ± 84	143 ± 37	233 ± 76	201 ± 53	166 ± 99	8.0 ± 3.8	5.0 ± 1.7	3.8 ± 2.1
BK	12565 ± 2356	536 ± 156	464 ± 90	148 ± 42	247 ± 81	201 ± 54	166 ± 98	8.0 ± 3.8	5.3 ± 1.7	4.0 ± 2.2
*RH vs BK (RB)*	*ns*	***	**	*ns*	*	*ns*	*ns*	*ns*	*	*ns*
Soil * RB	*ns*	**	*ns*	*ns*	*ns*	*ns*	*ns*	*ns*	*ns*	*ns*

Mean values ± Standard Errors; n=5. Soil (S), RH vs BK (RB) and their interaction (S x RB) were compared by two-way ANOVA. Tukey's HSD *post hoc* test (* p<0.05; ** p<0.01; *** p<0.001; ns, not significant). Different lowercase letters within each column indicate significant differences (p<0.05).

**Table 6:** Polyphenol content of fava beans samples cultivated on sandy-loam volcanic soil (VS), clay red soil (RS), pure fluvial sand (S100), sand mixed with green compost (70:30 v:v; S70C30), pure MMS-1 simulant (R100), MMS-1 amended with green compost (70:30 v:v; R70C30), expressed as µg/g of dry weight.

Polyphenols	VS	RS	S100	S70c30	R100	R70c30
Catechin	53.9 ± 0.8^c^	75.8 ± 0.4^a^	63.2 ± 4^b^	41 ± 0.7^e^	73.9 ± 2.1^a^	46.5 ± 0.4^d^
*p*-hydroxybenzoic acid	29.7 ± 0.05^a^	30.6 ± 3.7^a^	6.5 ± 0.6^b,c^	3.2 ± 0.05^c^	9.6 ± 1.2^b^	9.1 ± 0.8^b^
Epicatechin	95 ± 8.5^c^	91.9 ± 3.6^c^	164.6 ± 13.4^b^	377.5 ± 18.1^a^	143.8 ± 1.6^b^	387.9 ± 17.6^a^
Caffeic acid	88.3 ± 1.8^c^	55 ± 1.8^d^	117.9 ± 9.1^b^	68.4 ± 5.6^d^	145.7 ± 13.4^a^	63.7 ± 1.6^d^
Rutin	10.15 ± 0.3^d^	8.2 ± 0.2^d^	27 ± 1.8^b^	18.9 ± 0.3^c^	48.2 ± 4.2^a^	19.1 ± 0.1^c^
Apigenin-7-*O*-glucoside	35.6 ± 0.4^d^	40.6 ± 4.2^d^	96.5 ± 2.2^b^	84.8 ± 9.8^b,c^	120.1 ± 6.2^a^	80.8 ± 2.4^c^
*p*-Coumaric acid	24.6 ± 0.8^c^	21 ± 2.4^c^	43.2 ± 2.1^b^	44.7 ± 3.1^b^	46.4 ± 1.1^b^	51.7 ± 0.3^a^
Ferulic acid	7.1 ± 0.1^e^	9 ± 0.3^d^	13.1 ± 0.9^c^	15.8 ± 1.1^b^	24 ± 0.1^a^	10.7 ± 0.7^d^
Naringin	19.8 ± 2.8^d^	30.6 ± 1.2^c^	37.2 ± 0.2^b^	30.9 ± 2.5^c^	41.8 ± 1.6^b^	48.2 ± 1.5^a^
Myricetin 3-*O*-glucoside	5.6 ± 0.2^c^	7.6 ± 0.4^b^	6.4 ± 0.1^b,c^	5.9 ± 0.1^c^	14.9 ± 1.4^a^	6.3 ± 0.1^b,c^
Myricetin	6.7 ± 0.9^b^	6.1 ± 0.9^b,c^	3.9 ± 0.6^d^	4 ± 0.2^d^	4.6 ± 0.5^c,d^	11.9 ± 0.4^a^
Total	376.4 ± 7.5^d^	376.6 ± 8.1^d^	579.5 ± 16.2^c^	673 ± 3.9^b^	695 ± 5.1^b^	735.8 ± 25^a^

Data are shown as mean ± SD. Different lowercase letters indicate differences between the samples in the same raw assessed by One-way ANOVA and Tukey's *post hoc* tests (p<0.05).

The beneficial role of compost in the enhancement of the mineral nutrient quality of fava bean seeds is much more evident in terms of the nutrient content (**Table 3**). For instance, the fava seeds produced by a R70C30 plant could feed an astronaut with a total of total of 222 mg of essential nutrients such as K, N, S, P, Mg, Ca and Na (value obtained by the sum of their contents) and 667 μg of other healthy nutrients such as Na, Fe, Zn, B, Mn and Cu (**Table 3**); these values are much higher than those assessed with the pure R100 Mars simulant (i.e., 133 mg of summed K, N, S, P, Mg, Ca and Na, and 515 μg of summed Na, Fe, Zn, B, Mn and Cu).

The results of polyphenol analysis of fava bean samples grown in different soil types are reported in **Table 6**. The concentration of total polyphenols of these ranged from 376 to 735 µg/g. Epicatechin (91-388 µg/g) was the most abundant compound, followed by caffeic acid (55-146 µg/g), catechin (41-75.8 µg/g), and p-coumaric acid (21-52 µg/g), accounting approximatively for 70% of the total polyphenols. Comparing the fava beans grown in volcanic sandy-loam soil (VS) and red clay soil (RS), there were no significant differences in most of phenolic compounds, except for catechin (75.8 ± 0.4 µg/g), ferulic acid (9 ± 0.3 µg/g), naringin (30.6 ± 1.2 µg/g), and myricetin 3-O-glucoside (7.6 ± 0.4 µg/g), whose concentrations were significantly higher in RS (p<0.05). Both samples exhibited the highest value of p-hydroxybenzoic acid (on average 30 µg/g); however, they had the lowest total polyphenol content compared to the other samples. Interestingly, the samples cultivated on the fluvial sand and Mojave Mars regolith simulant mixed with the green compost, namely S70C30 and R70C30, showed a content of epicatechin 2.3- and 2.7-fold higher than the counterparts S100 and R100, respectively. Conversely, these samples exhibited 1.7- and 2.3- fold higher caffeic acid content than R70C30 and S70C30, respectively. Among all the samples, R70C30 had the highest concentration of epicatechin (387.9 ± 17.6 µg/g), p-coumaric acid (51.7 ± 0.3 µg/g), naringin (48.2 ± 1.5 µg/g), and myricetin (11.9 ± 0.4 µg/g). Similarly, the sample R100 contained highest level of caffeic acid (145.7 ± 13.4 µg/g), rutin (48.2 ± 4.2 µg/g), apigenin 7-O-glucoside (120.1 ± 6.2 µg/g), and ferulic acid (24 ± 0.1 µg/g).

**Figure 5:**
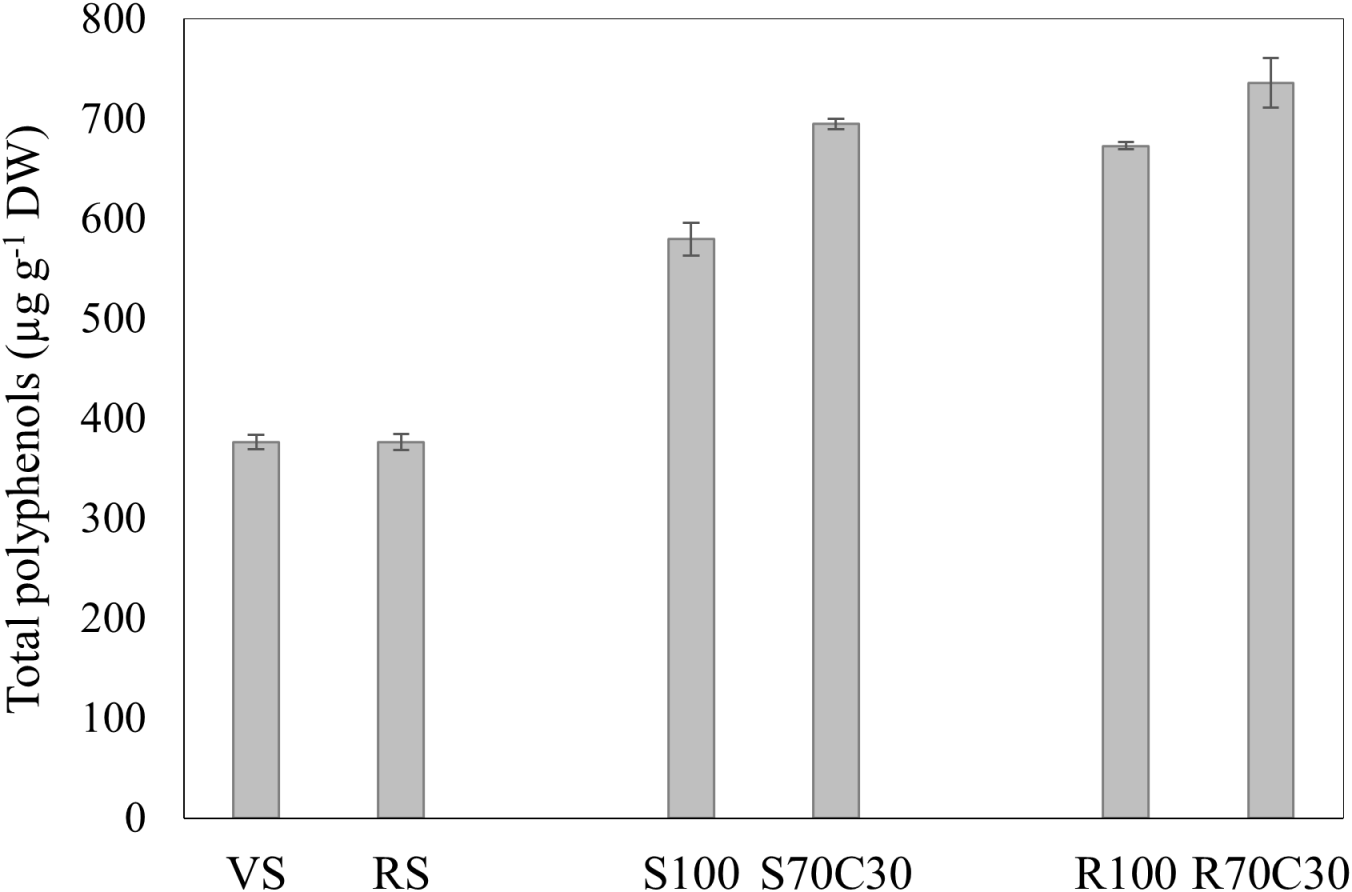
Total polyphenols (µg g^-1^ DW) in seeds of faba bean cv. 'Sfardella' grown on volcanic sandy-loam soil (VS), red clay soil (RS), pure fluvial sand (S100), sand mixed with green compost (70:30 v:v; S70C30), pure MMS-1 simulant (R100), MMS-1 amended with green compost (70:30 v:v; R70C30).

To conclude, as shown in **Figure 5**, the mixture of soils with green compost and the cultivation on Mojave Mars regolith simulant boosted the total polyphenol content of fava beans. Among the samples, fava seeds from R70C30 emerged as the most promising product exhibiting 1.4-fold higher total polyphenols than the other samples (735.8 ± 25 µg/g), followed by R100 and S70C30, which showed similar concentrations (p>0.05). Moreover, the cultivation on volcanic sandy-loam soil and red clay soil resulted in a low polyphenol content, with no statistically significant differences between them (p>0.05).

### 3.4 Chemical characterization of cultivation substrates after fava bean cultivation

The organic C, total N, and S concentrations, and the C/N ratio in the various substrates (separated in RH and BK soils at the plant sampling) are reported in **Supplementary Table S4**. The compost-amended growing media (R70C30 and S70C30) showed significantly higher concentrations of these elements than terrestrial soils (VS and RS) and pure substrates (R100 and S100); no statistically significant differences were found between RH and BK soils (**Supplementary Table S4**). We also evaluated the percentage variance (%) of C, N, and S contents after fava bean growing cycle (**Supplementary Figure S1**), compared with the contents assessed after potato growing cycle (Caporale et al., 2024). It is noteworthy that the content of the three elements significantly raised in the terrestrial soils and non-amended substrates, owing to the intense rhizosphere activity and bacterial symbiosis with a Fabaceae catch crop such as fava bean. For instance, in the pure Mars simulant (R100) the organic C, N, and S content increased by 52%, 19%, and 27%, respectively. The same compounds were much lower after fava bean cultivation in the compost-amended growing media, since part of the organic matter provided by the compost underwent to mineralization over time, which may have led to a partial release of these elements as volatile gases (**Supplementary Figure S1**).

The bioavailability of the main nutrients in the different substrates was assessed by two single extractions (1M NH4NO3 - easily bioavailable fractions: **Table 4**; and 0.05M EDTA at pH 7 - potentially bioavailable pools: **Table 5**). In both cases, a statistically different bioavailability of key nutrients was found among the growing media; compost-enriched substrates showed a similar or higher nutrient bioavailability than terrestrial soils (on average, 2.0-fold higher in **Table 4** excluding B; 1.7-fold higher in **Table 5**), and much greater than non-amended substrates (on average, 2.3-fold higher in **Table 4**; 2.5-fold higher in **Table 5**). Interestingly, the bioavailability of nutrients in the MMS-1-based substrates (R100 and R70C30) was much higher (4.9-fold the easily bioavailable fractions: **Table 4**; 2.7-fold the potentially bioavailable pools: **Table 5**) than sand-based ones (S100 and S70C30), which is basically due to the adsorbing surface of the zeolite (~12%) and clay (~2.5%) minerals in the MMS-1 simulant. We also found statistically significant variations between RH and BK soils for key elements of plant nutrition, such as K, Mg, P, Mn, Cu, and Zn (**Tables 4** and **5**), due to a diverse intensity of biological processes between RH and BK soils. The interaction between the factors of Soil (S) × RH vs. BK (RB) was significant (p < 0.05) for K, Fe, Mn, and Cu (**Table 4**), and only for K (**Table 5**). The sum of all the easily bioavailable fractions of nutrients (extracted by NH4NO3, a salt with weak acid hydrolysis) was approximately the 22% than the sum of all the potentially bioavailable nutrient fractions (extracted by EDTA, a complexing reagent) (**Tables 4** and **5**).

We also determined the percentage variance (%) of easily (**Supplementary Figure S2**) and potentially (**Supplementary Figure S3**) bioavailable fractions after the fava bean growing cycle, in comparison with the fractions assessed after the potato growing cycle (Caporale et al., 2024). From this assessment, we noted that the easily bioavailable fractions of the nutrients basically tended to decrease (in all the substrates) in favor of the potentially bioavailable fractions, which may suggest an increasing and more stable interaction of the main nutrients (also provided by fertigation) with the mineral and organic adsorbing surfaces of all the substrates over time. For instance, the easily bioavailable fractions decreased by 35% in S100 (on average for all the nutrients), 28% in S70C30 and 24% in R100; on the other hand, the potentially bioavailable fractions increased by 22% in S100 (on average for all nutrients), 23% in S70C30, 41% in R100, and 34% in R70C30.

We measured the pH (**Supplementary Figure S4A**) and EC (**Supplementary Figure S4B**) of the substrates after fava bean plant growth as well, two factors which can strongly affect nutrient bioavailability. The addition of compost to the simulant or sand mitigated their alkalinity (**Supplementary Figure S4A**), although the pH of these substrates remained above 8.0, which may have limited the bioavailability of the main nutrients. We did not find statistically significant variations between the pH values of RH vs. BK soils. The EC of the amended simulant or sand (S70C30 and R70C30) was significantly higher than the EC values in non-amended substrates (S100 and R100). No statistically significant differences were observed between RH and BK soils, apart from a significant decrease in rhizo soil of VS (**Supplementary Figure S4B**).

## 4 Discussion

Our results showed that the type of growth substrate significantly impacts the photosynthetic ability of fava bean plants at both 35 and 62 DAT. Specifically, photosynthetic rates were higher during the vegetative stage and decreased during the flowering and pod-filling stages across all substrates. This decline can be attributed to a shift in plant metabolic priorities. During the vegetative stage (35 DAT), the plant focuses on active leaf expansion and biomass accumulation, maximizing photosynthetic capacity. As the plant transitions to reproductive stages (62 DAT), resources such as carbohydrates are redirected from leaves to developing flowers, pods, and seeds, reducing the overall photosynthetic efficiency and stomatal conductance. Additionally, leaf aging during these stages likely contributes to the decline in photosynthetic rates (Taiz et al., 2015). The two-way ANOVA confirmed significant effects of both treatment and date, as well as their interaction, on NP, gs, and E, indicating that the decline in photosynthetic activity during growth mainly depended on substrate type.

The largest decreases in photosynthesis and stomatal conductance were observed in RS and R100 (especially at 62 DAT) substrates, and also R70C30 for gs. RS (clay red soil) may be unsuitable for growing fava beans in pots due to its tendency to compact and retain excess water, leading to poor drainage and increased risk of root rot (Athow, 2022; Chilakala et al., 2022).

However, the highest transpiration rate was observed only in plants grown on R100 (regolith alone), especially towards the end of the cycle (62 DAT), implying that the substrate caused the greatest water loss, thereby increasing the risk of tissue dehydration. Regolith lacks water retention, nutrients, and organic matter, thus it restricts plant growth. Consequently, the elevated transpiration rate in R100 plants may limit the feasibility of using regolith alone as a substrate for future space cultivation. This is supported by the reduced aerial biomass observed in R100 during our experiment, as well as a reduction in net-photosynthesis at both 35 and 62 DAT.

When regolith was mixed with compost (R70C30), transpiration decreased by 35%, while photosynthesis increased by 20%. These results are consistent with findings by Caporale et al. (2023) for potato plants. In both potato and fava bean plants, compost likely played a key role in retaining more water, owing to its well-known properties that enhance the soil's water-holding capacity (Adugna, 2016).

Our data are supported by photochemical analyses. In fact, both ΦPSII and ETR were significantly affected by treatment, date, and their interaction, suggesting a diminished ability to convert light into energy at the reaction centers in the photosystems, particularly in R100. The lack of many significant differences in ETR at 35 DAT, despite treatment effects being already evident in other photochemical parameters, is likely due to the relatively uniform canopy development at this early growth stage, when light use efficiency was not yet strongly differentiated among treatments. However, the Fv/Fm index remained unchanged and proximal to 0.8 across treatments, indicating that the photosystem II was not compromised by the growth in any substrate. Fv/Fm is a reliable indicator of plant health, with values around 0.8 reflecting optimal conditions (Björkman and Demmig, 1987), while lower values signal unfavourable conditions for the photosynthetic machinery, often due to stress (Baker and Rosenqvist, 2004). Only at 62 DAT, RS plants exhibited a significant yet modest decline in Fv/Fm, likely due to insufficient thermal dissipation to provide effective photoprotection. Thermal dissipation, particularly through mechanisms like non-photochemical quenching (NPQ), helps to dissipate excess light energy as heat, preventing damage to the photosynthetic apparatus. If this process is insufficient the excess energy may overwhelm the system, leading to a reduction in photosystem efficiency, as indicated by the decrease in Fv/Fm. In RS plants, the thermal dissipation might not have been sufficient to protect the photosynthetic machinery, resulting in the observed decrease in Fv/Fm. This suggests that although some dissipation occurred, it was insufficient to maintain optimal photosynthetic function under the given conditions. Consistent with this, NPQ was significantly influenced by treatment, date, and their interaction, showing the highest values in regolith-based substrates (R100 and R70C30) at both sampling date, and the lowest in VS at 62 DAT and S100, especially at 35 DAT. This indicates that regolith-based substrates promoted a greater need for energy dissipation, whereas sand- and compost-based substrates allowed more efficient photochemistry with reduced reliance on non-photochemical quenching.

Interestingly, RS was the treatment that enhanced photosynthetic pigment production, likely as a compensatory mechanism to optimize light absorption and protect the photosynthetic apparatus from oxidative damage. This increase in pigment production is consistent with findings that plants enhance pigment content to improve photosynthetic efficiency under stress (Lichtenthaler and Babani, 2002; Havaux, 1996).

The leaf area and the biomass accumulation in the aerial part were greater in plants grown on volcanic soil (VS) and on the sand and compost mixture (S70C30) compared to the other substrates, confirming the benefit of organic matter known for fava bean. The growth on the pure Mars regolith simulant (R100) reduced the aerial biomass accumulation compared to VS and S70C30 and gave similar values to the other substrates; however, fava bean plants completed the growing cycle also in MMS-1, confirming that the negative results of the previous experiment on soybean grown on the same substrate indicate a specific sensitivity of *Glycine max* L. to this regolith simulant.

The addition of green compost (R70C30) strongly improved the seed production (+61.9% with respect to R100), giving the same productive performance as VS and S70C30, with no relevant effect on the plant leaf area and aerial biomass, hence with a parallel increase in the harvest index (implying a reduction in the inedible biomass percentage, hence of the waste to be disposed). These positive effects of compost were presumably due to the improvement of the above-mentioned physical and chemical features and the overall fertility of the regolith simulant, enhancing the plant performance, as observed in lettuce (Duri et al., 2020) and potato (Caporale et al., 2023b) plants grown on the same substrates.

Fava bean plants cv. 'Sfardella', grown in pot in unheated glasshouse, in winter-spring period, completed the seed-to-seed cycle and formed healthy seeds on all the tested substrates in 92 days, which is the expected time for the cultivation period, considering the positive effect on the earliness of the controlled climate conditions. In all the substrates, the seed production was lower than that expected for the genotype, based on technical data provided by the breeder, while the dry matter percentage of seeds reached the average values known for this genotype.

The amendment of MMS-1 simulant with compost enhanced the productivity of fava bean plants, the nutritional value of the fava seeds (in terms of concentrations and contents of mineral nutrients) and total polyphenols. However, the seeds of R100 plants had a similar nutritional quality to seeds produced on terrestrial or compost-amended substrates; hence, MMS-1 pure simulant allowed fava bean plants to produce a yield of high quality, but with a low productivity.

Despite Mars regolith simulant MMS-1 contains several essential nutrients for plant growth (Caporale et al., 2020), it is not able to fulfil the plant requirements. This is due to the absence of organic matter and related key nutrients such as N, P and S, but also to the low bioavailability of the other essential nutrients intimately occluded in the mineral lattices. The mix of MMS-1 simulant with a stable organic matter as compost was found to be a sustainable practice for creating a more dynamic environment where microbiota and humified organic matter may interact with the mineral moiety (Duri et al., 2025), turning the nutrient-poor and alkaline Mars simulant into an efficient life-sustaining substrate equipped with enhanced physical, hydraulic, and chemical properties (Duri et al., 2022a; Caporale et al., 2023b). The organo-mineral interactions and the stabilization of exogeneous organic matter (from compost) by MMS-1 simulant minerals, such as Fe oxides, were recently studied by Giannetta et al (2023); Giannetta et al, (2025), since the accrual of organic matter in this mineral matrix is crucial to maintain the long-term fertility of these plant growing substrates over time and the survival and the healthiness of crops during the space missions. By sample fractionation in particulate (POM) and mineral-associated (MAOM) organic matter, and X-ray absorption spectroscopy (XANES and EXAFS), these authors found that ferrihydrite mediated exogenous organic matter stabilization in the regolith-based amended substrate (R70C30), while they observed the formation of Fe(III)-organic matter complexes only in the sand-based growing medium (S70C30). Understanding the mechanisms behind the formation of organo-mineral interactions can help improving sustainable space farming, since these biochemical processes - leading to the creation of a porous and highly dynamic environment - might strongly affect the bioavailability of nutrients.

Nutrient bioavailability in the rhizosphere is influenced by several soil properties, such as pH and EC, clay and organic matter contents, and is controlled by a wide range of dynamic processes (Adamo et al., 2024). Therefore, accurate extraction and quantification of bioavailable nutrient pools, is essential for understanding the mobility of the nutrients in the growing substrate-plant system. Our bioavailability extractions (by 1 M NH4NO3 and 0.05 M EDTA at pH 7) revealed that all substrates supplied bioavailable fractions of essential nutrients to fava bean plants. However, without an adequate compost amendment, both the Mars simulant and fluvial sand fall short of meeting the plants' nutritional needs unless supplemented through fertigation. By analysing the dynamics of nutrient bioavailability over time (by comparing the bioavailable fractions assessed after fava bean cultivation and those after the previous experiment with potato, published in Caporale et al., 2024), we observed that the easily bioavailable nutrient fractions generally declined across all substrates, whereas the potentially bioavailable fractions increased. This trend suggests a strengthening and more stable interaction of key nutrients - including those supplied through fertigation - with the mineral and organic adsorption surfaces of the substrates over time. Therefore, it is clear that the addition of composted organic matter to a Mars regolith simulant is not enough to create an optimal substrate for space farming. Moreover, it is also essential to use this medium in consecutive plant-growing cycles to progressively enhance its physical, chemical, and biological fertility, thereby ensuring consistent crop productivity and quality over time. Regarding the characterization of polyphenols, the samples analyzed in this study exhibited a phenolic profile similar to those previously reported (Valente et al., 2018; Johnson et al., 2021), with quantitative differences depending on cultivation conditions. Among the samples, faba bean seeds grown on R70C30 showed the highest total polyphenol content, followed by those grown on R100 and S70C30. This suggests the efficacy of green compost in enhancing the production of secondary plant metabolites, particularly p-coumaric acid (Verrillo et al., 2021).

## 5 Conclusions

Within a research project aiming at identifying regolith-based substrates suitable for consecutive cultivation cycles of candidate crops, to develop a sustainable BLSS for Mars surface, we assessed the response of fava bean plants to greenhouse pot cultivation on the Martian regolith simulant MMS-1 compared to different terrestrial substrates, in terms of plant growth and productivity, seed quality, and change of substrate fertility.

Our results demonstrated that consecutive plant-growing cycles on the same Mars regolith simulant-based substrates can improve their agronomical performance, lowering the pH, increasing mineral weathering, enhancing nutrient release/bioavailability, promoting the aggregation of medium-fine particles and the formation of an efficient porous system, and likely improving microbiota diversity and abundance. The assessment of nutrient bioavailability dynamics over time (before and after the fava bean growing cycle) indicated a progressive increase of potentially bioavailable nutrient fractions, hence, the formation of more stable interactions of key nutrients with the mineral and organic phases of the substrates.

In general, the growth on the pure MMS-1 Martian regolith simulant reduced all the measured physiological and growth parameters and the seed productivity compared to terrestrial substrates, despite the supply of nutrients through fertigation. However, the addition of green compost mitigated the alkalinity and improved physicochemical properties, nutrient bioavailability, and fertility of the regolith simulant, resulting in positive effects on the seed production, the harvest index and the nutritional seed quality of fava bean, in line with the results observed in terrestrial soils, particularly volcanic soils.

Besides crop succession on the same Martian simulant-based substrates, further research reasonably needs to be conducted under the space altered gravity conditions, which can strongly affect plant physiology, plant growth, productivity and quality, and substrate characteristics over time.

## Data Availability

The original contributions presented in the study are included in the article/**Supplementary Material**. Further inquiries can be directed to the corresponding author.
