# Supplementary material for: Fava bean plant performance and fertility dynamics in Mars regolith simulant-based substrates for space farming
Source: Front Plant Sci. 2025 Oct 13;16:1676285. doi: 10.3389/fpls.2025.1676285 (PMC12556254; doi:10.3389/fpls.2025.1676285)
Supplement: Supplementary file 1 [file DataSheet1.docx]

Supplementary Material

## Supplementary Figures

**Supplementary Figure 1.** Percentage variance (%) of C, N and S contents after fava bean growing cycle, compared with the contents assessed after potato growing cycle (Caporale et al., 2024), in volcanic soil (VS), red soil (RS), pure fluvial sand (S100), sand mixed with green compost (70:30 v:v; S70C30), pure MMS-1 simulant (R100), MMS-1 amended with green compost (70:30 v:v; R70C30).


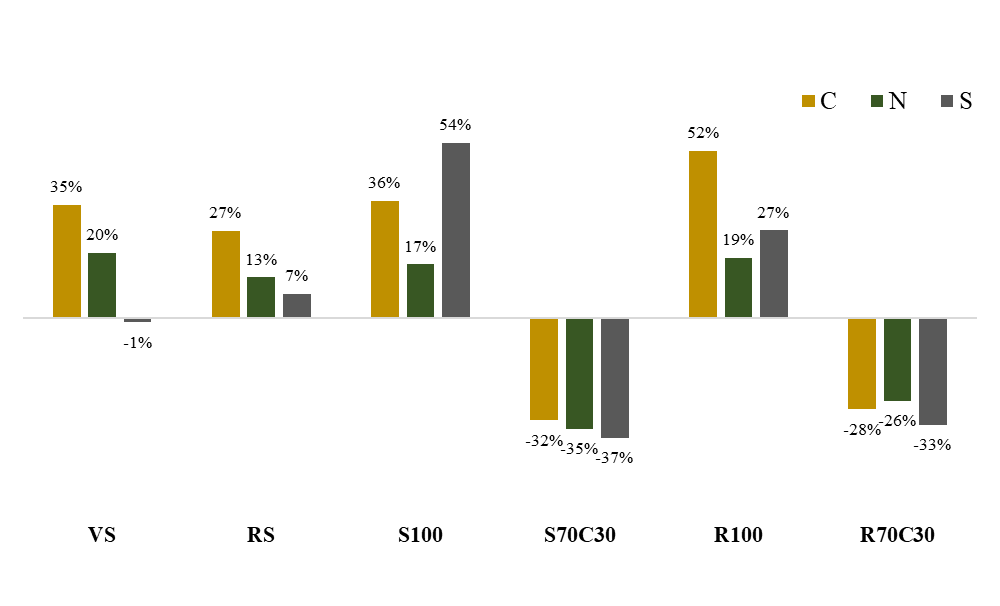


**Supplementary Figure 2.** Percentage variance (%) of concentrations of main macro and micronutrients extracted by 1M NH4NO3 after fava bean growing cycle, from volcanic soil (VS), red soil (RS), pure fluvial sand (S100), sand mixed with green compost (70:30 v:v; S70C30), pure MMS-1 simulant (R100), MMS-1 amended with green compost (70:30 v:v; R70C30), in comparison with the concentrations extracted by the same reagent after potato growing cycle (Caporale et al., 2024).


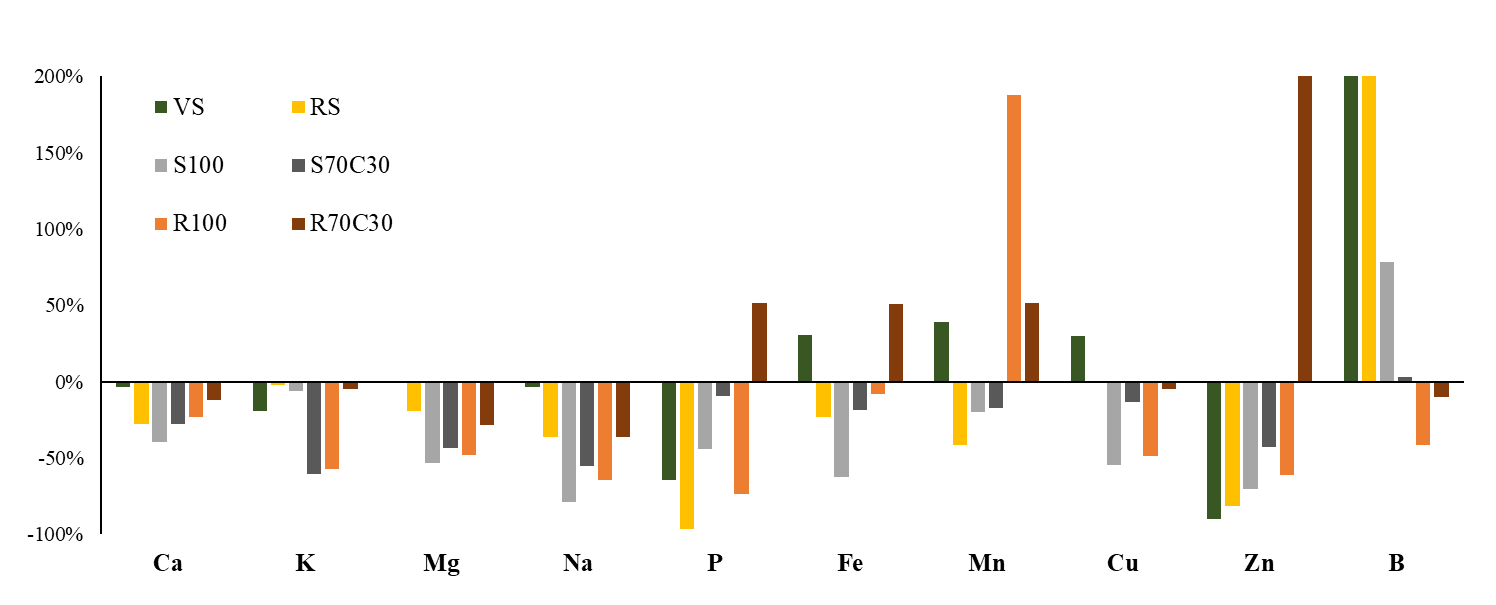


**Supplementary Figure 3.** Percentage variance (%) of concentrations of main macro and micronutrients extracted by 0.05M EDTA (buffered at pH 7) after fava bean growing cycle, from volcanic soil (VS), red soil (RS), pure fluvial sand (S100), sand mixed with green compost (70:30 v:v; S70C30), pure MMS-1 simulant (R100), MMS-1 amended with green compost (70:30 v:v; R70C30), in comparison with the concentrations extracted by the same reagent after potato growing cycle (Caporale et al., 2024).


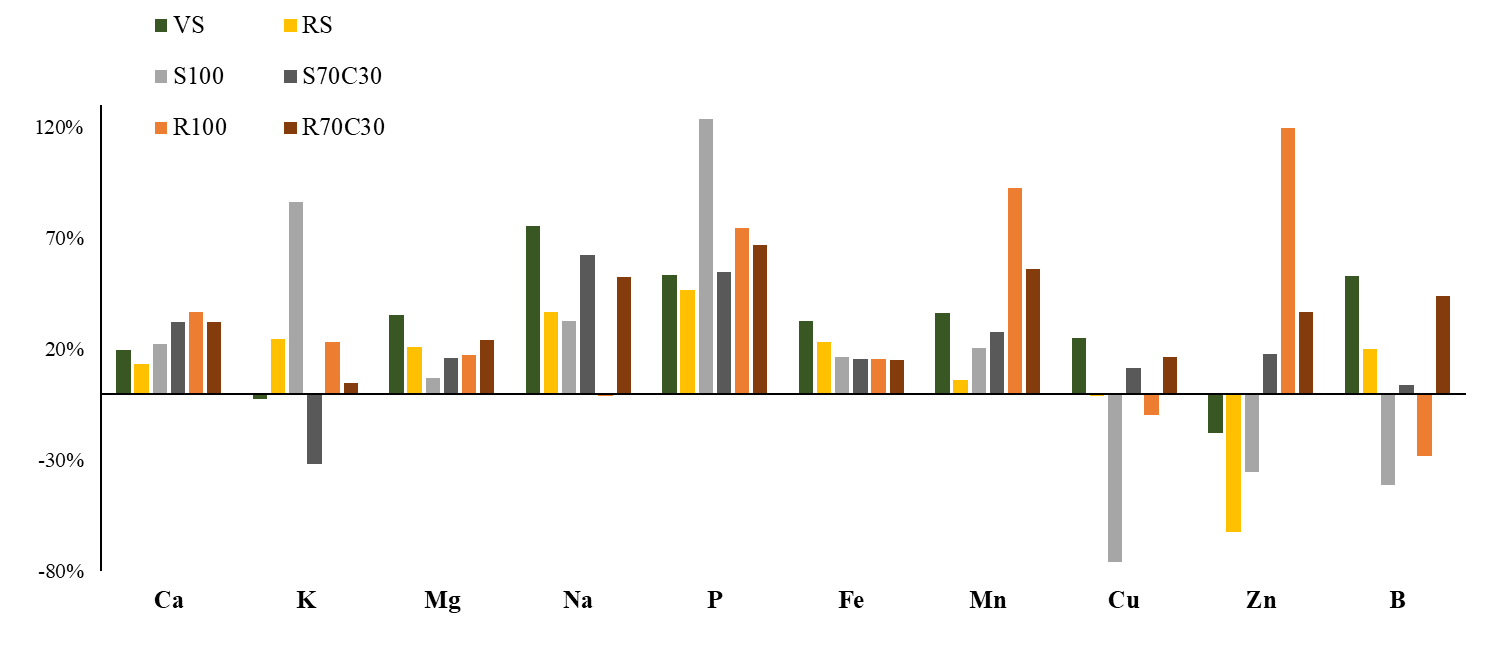


**Supplementary Figure 4.**

## 2 Supplementary Tables

**Supplementary Table 1.** Retention times of identified polyphenols by HPLC UV/VIS.

| Phenolic compound | Retention time (min) |
| --- | --- |
| Catechin | 8 |
| *p*-hydroxybenzoic acid | 10.5 |
| Epicatechin | 11 |
| Caffeic acid | 11.9 |
| Apigenin 7-O-glucoside | 14.9 |
| *p*-Coumaric acid | 15.3 |
| Rutin | 15.9 |
| *Trans*-ferulic acid | 16.5 |
| Naringin | 19 |
| Myricetin 3-O-glucoside | 21 |
| Myricetin | 22.1 |
|  |  |

**Supplementary Table 2.** Effect of treatment (T), date (D), and their interaction (T × D) on gas-exchange and photochemistry parameters (NP, gs, E, ΦPSII, ETR, Fv/Fm, NPQ).

|  | NP  (µmol CO_2_ m^-2^s^-1^) | gs  (mmol CO_2_ m^-2^s^-1^) | E  (mol H_2_O m^-2^s^-1^) | ΦPSII | ETR | FvFm | NPQ |
| --- | --- | --- | --- | --- | --- | --- | --- |
| Treatment | *** | *ns* | ***** | *** | *** | ** | *** |
| Data | *** | *** | *ns* | ** | *** | *ns* | ** |
| *T x D* | *** | *** | ***** | **** | *** | **** | *** |

*Treatment, Data and their interaction (T × D) were compared by two-way ANOVA, Tukey’s HSD post hoc test (* p*≤*0.05; ** p*≤*0.01; *** p*≤*0.001; ns: not significant).*

**Supplementary Table 3**. Concentrations (g kg-1 DW and mg kg-1 DW) of main nutrients in grains of fava bean cv. Sfardella, grown on sandy-loam volcanic soil (VS), clay red soil (RS), pure fluvial sand (S100), sand mixed with green compost (70:30 v:v; S70C30), pure MMS-1 simulant (R100), MMS-1 amended with green compost (70:30 v:v; R70C30). Data are expressed as mean values of 5 replicates.

|  | VS | RS | S100 | S70C30 | R100 | R70C30 | Sig. |
| --- | --- | --- | --- | --- | --- | --- | --- |
| g kg^-1^ DW |  |  |  |  |  |  |  |
| C | 414 | 397 | 414 | 439 | 427 | 411 | ns |
| N | 41.2 a | 39.2 ab | 30.2 c | 34.8 bc | 33.0 c | 32.2 c | *** |
| K | 12.6 cd | 11.9 d | 13.3 bc | 14.3 b | 15.7 a | 11.8 d | *** |
| S | 4.2 a | 3.6 ab | 2.9 bc | 2.9 bc | 2.6 bc | 2.5 c | *** |
| P | 2.1 ab | 2.3 a | 1.8 b | 2.2 ab | 2.0 ab | 1.8 b | ** |
| Mg | 1.0 b | 0.9 b | 1.0 b | 1.2 a | 1.1 a | 0.9 b | *** |
| Ca | 0.7 bc | 0.9 a | 0.8 ab | 0.7 bc | 0.9 ab | 0.7 c | *** |
|  |  |  |  |  |  |  |  |
| mg kg^-1^ DW |  |  |  |  |  |  |  |
| Na | 91.1 a | 78.4 a | 52.2 b | 49.7 b | 86.9 a | 70.8 ab | *** |
| Fe | 50.0 b | 34.7 c | 45.8 b | 45.0 b | 59.2 a | 36.8 c | *** |
| Zn | 28.2 a | 23.6 b | 15.6 d | 22.7 bc | 21.0 bc | 20.3 c | *** |
| B | 9.8 b | 9.1 b | 8.7 b | 8.7 b | 30.0 a | 10.7 b | *** |
| Mn | 8.3 bc | 10.3 a | 9.3 ab | 7.6 c | 9.8 a | 7.2 c | *** |
| Cu | 10.6 a | 8.6 b | 7.8 bc | 6.5 d | 7.5 cd | 4.2 e | *** |

*For the sake of clarity, this wide table shows only the mean values (n=5), not followed by standard errors. Different letters within each row indicate significant differences according to one-way ANOVA. Tukey’s HSD post hoc test (* p<0.05; ** p<0.01; *** p<0.001; ns: not significant).*

**Supplementary Table 4.** Concentrations (g kg-1 and mg plant-1 DW) of organic C, total N and S, C/N ratio in sandy-loam volcanic soil (VS), clay red soil (RS), pure fluvial sand (S100), sand mixed with green compost (70:30 v:v; S70C30), pure MMS-1 simulant (R100), MMS-1 amended with green compost (70:30 v:v; R70C30), separated in rhizo (RH) and bulk (BK) soils after fava bean plant growth. Data are expressed as mean values ± standard errors (n = 5).

|  | C | N | C/N | S |
| --- | --- | --- | --- | --- |
|  | g kg^-1^ DW | g kg^-1^ DW |  | mg kg^-1^ DW |
| VS | 24.6 ± 1.2 b | 1.84 ± 0.09 bc | 13.4 ± 0.7 | 816 ± 94 abc |
| RS | 19.3 ± 0.4 bc | 1.61 ± 0.05 c | 12.0 ± 0.3 | 712 ± 68 bc |
| S100 | 3.0 ± 0.4 c | 0.21 ± 0.03 c | 14.3 ± 3.8 | 139 ± 19 c |
| S70C30 | 49.5 ± 7.4 a | 3.68 ± 1.34 ab | 13.5 ± 2.6 | 1260 ± 416 ab |
| R100 | 3.2 ± 0.4 c | 0.35 ± 0.05 c | 9.0 ± 2.4 | 159 ± 49 c |
| R70C30 | 53.9 ± 11.6 a | 4.27 ± 0.72 a | 12.6 ± 0.6 | 1484 ± 404 a |
| *Soil (S)* | ***** | ***** | *ns* | ***** |
|  |  |  |  |  |
| RH | 27.4 ± 12.4 | 2.20 ± 1.13 | 12.5 ± 1.8 | 878 ± 415 |
| BK | 23.7 ± 8.4 | 1.79 ± 0.63 | 13.2 ± 2.7 | 645 ± 193 |
| *RH vs BK (RB)* | *ns* | *ns* | *ns* | *ns* |
|  |  |  |  |  |
| *S x RB* | *ns* | *ns* | *ns* | *ns* |

*Soil (S), RH vs. BK (RB) and their interaction (S × RB) were compared by two-way ANOVA, Tukey’s HSD post hoc test (** p<0.01; *** p<0.001; ns: not significant). Different lowercase letters within each column indicate significant differences (p<0.05).*
